# A time of change at *Thrombosis Journal*

**DOI:** 10.1186/1477-9560-10-8

**Published:** 2012-05-29

**Authors:** Hugo ten Cate, Yukio Ozaki

**Affiliations:** 1 University Medical Center, UNS 50/box 8, PO Box 616, Maastricht, Limburg, 6200 MD, Netherlands; 2 Department of Laboratory Medicine, University of Yamanashi, Shimokato 1110, Chuo, Yamanashi, 409-3898, Japan

## Abstract

Thrombosis and hemostasis related disease have a heavy burden in cardiovascular disease and it is important to have a journal where research into this can be accessed by all.

## 

At the start of our Editorship we wanted to look back and reflect upon the journal’s successes to date, but it is also important to look forward! The future of *Thrombosis Journal* is taking shape and we would like to invite all members of the community to contribute to the further development of the journal to best suit the community’s needs.

The mission statement of *Thrombosis Journal* was clearly stated by the founding Editor, Dr. Raul Altman, a highly respected and internationally known investigator in the area of thrombosis and haemostasis. On the journal’s website [1] he says: "Everyone should have equal access to medical literature. Thrombosis is the most significant cause of disability and death in the world. *Thrombosis Journal*, as an open access publication, makes it possible for anyone in the world to read high-quality research in this important area”.

In a nutshell, this statement summarizes two important features. Firstly, open access is being increasingly recognised as a universal human right, in this particular case the right of clinicians and scientists to unrestricted access to new scientific data emerging from our field of interest. Secondly, the fact that thrombosis and hemostasis related diseases are the most important burden of cardiovascular disease and death worldwide [2,3]! Thrombosis (arterial or venous) is a global problem of major significance and while there are many related biomedical journals that either carry the topic as a main issue (e.g., *Journal of Thrombosis and Haemostasis*), or devote significant space to thrombosis and haemostasis (e.g., *Blood*), the “thrombosis” scope is so wide that there is ample need for additional forums. In short, our mission statement remains unchanged, we aim to support the community through the dissemination of the freely accessible original research articles published in the journal. We consider open access a critical element in the dissemination of knowledge and research data throughout the world. In the field of thrombosis (and hemostasis) many developments are relevant, not only for the developed world, but also for those in developing nations where access to subscription journals may be limited, mainly because of financial constraints. As *Thrombosis Journal* charges its authors to publish articles (discretionary waivers are considered on a case by case basis for authors unable to pay), the strength of the journal is that it can be accessed by anyone, anywhere, without charge. This makes it a highly competitive journal in terms of readership, throughout the world. This is also illustrated by the “Highly accessed” papers indicated on the *Thrombosis Journal* website and indeed, some of these papers are also well cited, including a review authored with some of my colleagues [4] that has been cited 49 times and a research article by Borna *et al.*[5] 53 times. An essential element is, of course, the fact that all *Thrombosis Journal* publications are immediately included in PubMed and PubMed Central.

We acknowledge that potential authors can experience pressure from their employers, grant providers, etc., to publish in journals with a high impact factor. While we recongnize that we are still small in terms of such quality indicators, we will be working closely with the journal’s publisher, BioMed Central, to ensure we are attracting more publications of sufficient interest with aim of achieving an impact factor for the journal. Dr Yukio Ozaki and I were put forward by Dr. Altman as his successors, offering him the chance to take a small step back from his very busy working life. We are taking over with the strong belief that we will be able to further strengthen *Thrombosis Journal* as an open access journal that matters!

For this reason we urge you to consider us when submitting your next original work in the entire area of thrombosis and hemostasis research, case reports of interest, and review articles. Since we are an online journal there are no limitations on the numbers of figures or other illustrations or colors, which creates more flexibility. We also encourage initiatives for special issues or series of articles on any topic that fits the journal. Moreover, we would like to encourage a global scope, meaning that we encourage colleagues from anywhere to consider our journal for their publications. At the same time opening up does not mean becoming less critical of the contents of submissions. As before, we aim to accept papers that may not have high interest levels but are scientifically sound and contribute to the field of research.

In aiming for a global, open access journal for the entire community dealing with thrombosis and hemostasis related disorders (also including cardiovascular diseases with a thrombotic origin, such as myocardial infarction and stroke), we take the baton from Raul Altman. In addition to his research, Dr Altman is also known for his continuous efforts to provide high quality education in thrombosis and hemostasis throughout South America. We feel, as a final remark, that *Thrombosis Journal* could also contribute to education and therefore we welcome the introduction of a “In the clinic” series of reviews providing practical answers to situations encountered in thrombosis and hemostasis clinics. We invite you to submit any manuscripts that would fall under this category to the *Thrombosis Journal*.
